# Oral mucositis associated with anti-EGFR therapy in colorectal cancer: single institutional retrospective cohort study

**DOI:** 10.1186/s12885-018-4862-z

**Published:** 2018-10-05

**Authors:** Satoshi Dote, Shoji Itakura, Kohei Kamei, Daiki Hira, Satoshi Noda, Yuka Kobayashi, Tomohiro Terada

**Affiliations:** 1 Department of Pharmacy, Kyoto-Katsura Hospital, 17, Yamadahiraocho, Kyoto-shi Nishikyo-ku, Kyoto, 615-8256 Japan; 2 Department of Pharmacy, Shiga University of Medical Science Hospital, Seta Tsukinowa-cho, Otsu, Shiga 520-2192 Japan; 3 Department of Pharmacy, Japanese Red Cross Kyoto Daiichi Hospital, 15-749, Hommachi, Kyoto-shi Higashiyama-ku, Kyoto, 605-0981 Japan; 4 College of Pharmaceutical Sciences, Ritsumeikan University, Noji-higashi 1-1-1, Kusatsu, Shiga 525-8577 Japan

**Keywords:** Oral mucositis, Colorectal cancer, Panitumumab, Cetuximab, Anti-EGFR antibody, 5-fluorouracil

## Abstract

**Background:**

Chemotherapy-induced oral mucositis impairs the quality of life. The difference in severity of oral mucositis between different anti-epidermal growth factor receptor (EGFR) antibodies combined with cytotoxic drugs in colorectal cancer is unclear. The aim of this study was to investigate the differences in oral mucositis between panitumumab (Pmab) and cetuximab (Cmab) combined with 5-fluorouracil (5-FU).

**Methods:**

We conducted a retrospective cohort study. A total of 75 colorectal cancer outpatients treated with an anti-EGFR antibody combined with FOLFOX, FOLFIRI, or 5-FU/leucovorin as the first- to third-line treatment were included. The primary endpoint was the incidence of grade 2–3 oral mucositis. The secondary endpoint was the time to onset of oral mucositis. We also compared the incidence of toxicities of interest, skin toxicity, hypomagnesaemia and neutropenia, and time to treatment failure (TTF) between the two groups.

**Results:**

Thirty-two patients treated with Pmab and 43 patients treated with Cmab were evaluated. Patient characteristics were similar between the two groups. The incidence of grade 2–3 oral mucositis was significantly higher with Pmab than with Cmab (31.3% vs 9.3%, *P* < 0.05). Moreover, the incidence of grade 3 oral mucositis was significantly higher in patients treated with Pmab (18.8% vs 0%, *P* < 0.01). The mean (SD) cycles to onset of the worst oral mucositis was 3.0 (2.9) in the Pmab group and 2.3 (1.7) in the Cmab group (*P* = 0.29). Oral mucositis was characterized by glossitis and cheilitis. The incidences of other toxicities were the following (Pmab vs Cmab): grade 2–3 skin toxicity: 68.8% vs 74.4% (*P* = 0.61), grade 2–3 hypomagnesaemia: 9.3% vs 7.0% (*P* = 1.00), grade 3–4 neutropenia: 28.1% vs 37.2% (*P* = 0.46). The median TTF was not significantly different, i.e., 223 days vs 200 days (*P* = 0.39) for Pmab vs Cmab.

**Conclusions:**

Pmab-based chemotherapy resulted in significantly higher grades of oral mucositis compared with Cmab-based chemotherapy. The oral condition should be monitored carefully and early supportive care should be provided for patients treated with Pmab-based chemotherapy.

**Electronic supplementary material:**

The online version of this article (10.1186/s12885-018-4862-z) contains supplementary material, which is available to authorized users.

## Background

Oral mucositis refers to mucosal damage secondary to cancer therapy occurring in the oral cavity, and can be caused by both chemotherapy and radiotherapy [1–3]. Oral mucositis presents as erythema and/or ulceration of the oral mucosa. It is typically painful, requiring analgesics, leading to alteration in cancer therapy, risk for infection, and it impairs nutritional intake and quality of life [1–4].

Epidermal growth factor (EGF) regulates epithelial cell proliferation, growth, and migration, is present in biological fluids, including saliva, and plays an important role in maintaining the epithelial barrier and healing damaged mucosa [5]. Regarding the role of EGF in oral mucosa in oncology, salivary EGF levels have been reported to be associated with the severity of oral mucositis induced by radiation therapy [6, 7]. Furthermore, Kim et al. reported that recombinant human EGF oral spray improved mucotoxic regimen-induced oral mucositis in patients undergoing hematopoietic stem cell transplant [8]. Anti-epidermal growth factor receptor (EGFR) antibodies, panitumumab (Pmab) and cetuximab (Cmab), are widely used for patients with wild-type (WT) KRAS metastatic colorectal cancer [9–11]. The toxicities of anti-EGFR antibodies were characterized by skin toxicity [12, 13], infusion reaction [14–16], electrolyte imbalance [16–18], and interstitial pneumonia [16, 19]. It was also reported that the incidence of oral mucositis was 5 to 7% when an anti-EGFR antibody was used as monotherapy [20]. Interestingly, the incidence of oral mucositis was higher (all grades: about 30–40%, grade 3 or higher: approximately 10%) when the anti-EGFR antibody was used in combination with 5-fluorouracil (5-FU) [21–25], which is a well-known mucotoxic drug [26]. Although the mechanism of oral mucositis induced by the anti-EGFR antibody concomitant with 5-FU was not clarified, anti-EGFR therapy may deteriorate 5-FU-induced oral mucositis by interfering with the wound healing process due to blockage of EGF.

In Japan, board-certified oncology pharmacists provide pharmaceutical care for oncology outpatients [27, 28]. In our institute, board-certified oncology pharmacists routinely check the oral condition in outpatients as a part of pharmaceutical care. To date, in one comparative phase III study, there was not a significant difference in the incidence of oral mucositis between Pmab and Cmab used as monotherapy [20]. However, head-to-head studies comparing the incidence of oral mucositis between Pmab- and Cmab-combined with 5-FU have not been reported. Therefore, we conducted a retrospective cohort study to examine the incidence and severity of oral mucositis in patients who were receiving anti-EGFR antibodies concomitantly with 5-FU.

## Methods

### Study design

Study design was retrospective, single institutional cohort study. Eligible patients were metastatic colorectal cancer outpatients treated with an anti-EGFR antibody combined with FOLFOX: infusional 5-FU plus leucovorin (5-FU/LV) with the addition of oxaliplatin, FOLFIRI: 5-FU/LV with the addition of irinotecan, or 5-FU/LV as the first- to third-line treatment at Kyoto-Katsura Hospital (details of chemotherapy are shown in Additional file 1: Table S1). If the patients were treated with both Pmab and Cmab until the third-line of chemotherapy, we allocated the patient to the prior anti-EGFR antibody group. Exclusion criteria were 5-FU-free chemotherapy treatment and inpatients. The periods of recruitment and data collection (follow-up) from electronic medical records were January 1, 2012 to February 28, 2017 and January 1, 2012 to March 31, 2017, respectively. This study was performed in accordance with the Declaration of Helsinki and its amendments, and the protocol was approved by the Ethics Committee of Kyoto-Katsura Hospital (Approval number: 501).

### Procedures

We divided the eligible patients into two groups. The Pmab group included the patients treated with Pmab-based chemotherapy and the Cmab group included the patients treated with Cmab-based chemotherapy. The primary endpoint was the incidence of grade 2–3 oral mucositis (Table 1) in either groups. At our institute, board-certified oncology pharmacists and nurses catch adverse events by carefully interviewing while referring to the medical questionnaire answered by the patient and correctly recording the grade of toxicities in the electronic medical records. Oral mucositis was graded at each outpatient chemotherapy session by physicians, board-certified oncology pharmacists, and nurses. The secondary endpoint was the time to onset of oral mucositis, defined as the cycle when oral mucositis occurred after initiation of anti-EGFR therapy. A cycle was defined in biweekly intervals. If Cmab was given weekly, one cycle contained two Cmab infusions. The number of cycles at the first onset of any grade of oral mucositis and that at the onset of the worst grade of oral mucositis for each patient were compared between the two groups. Once anti-EGFR therapy was initiated, we counted the number of cycles regardless of whether anti-EGFR antibody administration was postponed or discontinued due to toxicity during chemotherapy. We also compared toxicities of interest between the two groups: the incidence of skin toxicity, neutropenia, and hypomagnesaemia, and time to treatment failure (TTF), which was defined as the time from treatment initiation to discontinuation for any reason, including disease progression, treatment toxicities, patient preference, or death. Any toxicities were graded according to the Common Terminology Criteria for Adverse Events (CTCAE) version 4.0.

**Table 1:** National Cancer Institute Common Terminology Criteria for Adverse Events v4.0 “Oral mucositis”

Grade 1	Grade 2	Grade 3	Grade 4	Grade 5
Asymptomatic or mild symptoms; intervention not indicated	Moderate pain; not interfering with oral intake; modified diet indicated	Severe pain; interfering with oral intake	Life-threatening consequence; urgent intervention indicated	Death

### Statistical analyses

Binary outcomes were compared with the Fisher’s exact test, and continuous outcomes with the unpaired Student’s t-test, and time-to-event data was compared by the log-rank test using the Kaplan-Meier method. JMP^®^9 software (SAS Institute, Japan) was used for all analyses and a *p*-value less than 0.05 was regarded as significant. The heterogeneity of the treatment effects on the primary endpoint was assessed for five pre-specified subgroups. Subgroups were based on history of diabetes and smoking status as risk factors for oral mucositis [3], and sex, treatment regimen, and line of treatment, which likely affect the incidence of oral mucositis. When patients underwent curative operations, such as hepatectomy or primary tumor resection, and radiotherapy during anti-EGFR antibody combined with 5-FU chemotherapy, the patient was treated as a censored case in the time-to-event analysis.

## Results

### Participants

Thirty-two patients were evaluated in the Pmab group and 43 patients were evaluated in the Cmab group. Patient characteristics are shown in Table 2. There were no significant differences in patient demographics between the two groups. Most patients were treated with the anti-EGFR antibody combined with 5-FU as first- or second-line chemotherapy and had a good performance status.

**Table 2:** Patient characteristics

	Pmab group *N* = 32	Cmab group *N* = 43	*P*-value
Sex			
Male	23 (72%)	30 (70%)	1.00
Age (years)	65.7 (9.0)	63.1 (12.4)	0.33
Body weight (kg)	58.1 (10.0)	57.9 (9.0)	0.91
Body surface area (kg/m^2^)	1.62 (0.17)	1.60 (0.16)	0.59
Performance Status (ECOG)			
0 / 1 / 2	15 (47%) / 16 (50%) / 1 (3%)	27 (63%) / 15 (35%) / 1 (2%)	0.39
Diabetes mellitus	8 (25%)	6 (14%)	0.25
Smoking status			
Never / former / current	8 (25%) / 15 (47%) / 9 (28%)	17 (40%) / 16 (37%) / 10 (23%)	0.42
Serum albumin level (g/dl)	3.64 (0.66)	3.74 (0.36)	0.43
Primary origin of tumors			
Rectal / colon / other	9 (28%) / 22 (69%) / 1 (3%)	22 (51%) / 18 (42%) / 3 (7%)	0.07
On the left side of the colon / on the right side of the colon^a^	21 (66%) / 11 (34%)	33 (78%) / 10 (23%)	0.31
Line of treatment			
1st / 2nd / 3rd	25 (78%) / 6 (19%) / 1 (3%)	38 (88%) / 4 (9%) / 1 (2%)	0.47
Combined regimen			
FOLFOX / FOLFIRI / LV5FU	22 (69%) / 9 (28%) / 1 (3%)	38 (88%) / 4 (9%) / 1 (2%)	0.10
Concomitant bolus 5-FU			
Presence / absence	32 (100%) / 0 (0%)	41 (95%) / 2 (5%)	0.50
Relative dose intensity in cycle 1 (%)	97.3 (7.8)	93.0 (21.6)	0.28
Cetuximab interval^b^			
Weekly / biweekly	-	13 (30%) / 30 (70%)	-

Data are expressed as mean (SD) and n (%).

^a^On the left side of the colon means descending colon, sigmoid colon, and rectum. On the right side of the colon means the cecum and ascending colon.

^b^Weekly means cetuximab administered weekly; the initial dose was 400 mg/m^2^ and the maintenance dose was 250 mg/m^2^. Biweekly means a 500-mg/m^2^ dose of cetuximab administered every other week.

### The incidence of oral mucositis

**Fig. 1:**
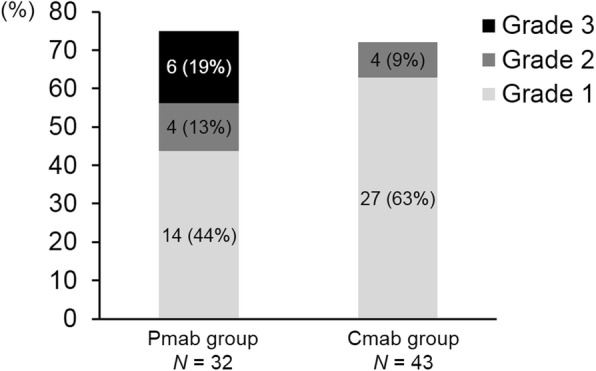
Primary endpoint: the incidence of grade 2–3 oral mucositis between the Pmab and Cmab groups. The number of patients (N) and the incidence of oral mucositis (%) of each grade are shown in the bar charts. The incidence of oral mucositis was the following (Pmab group vs Cmab group); All grades: 24 (75%) vs 31 (72%), *p* > 0.05. grade 2–3: 10 (31.3%) vs 4 (9.3%), *p* < 0.05. grade 3: 6 (18.8%) vs 0 (0%), *p* < 0.01

**Fig. 2:**
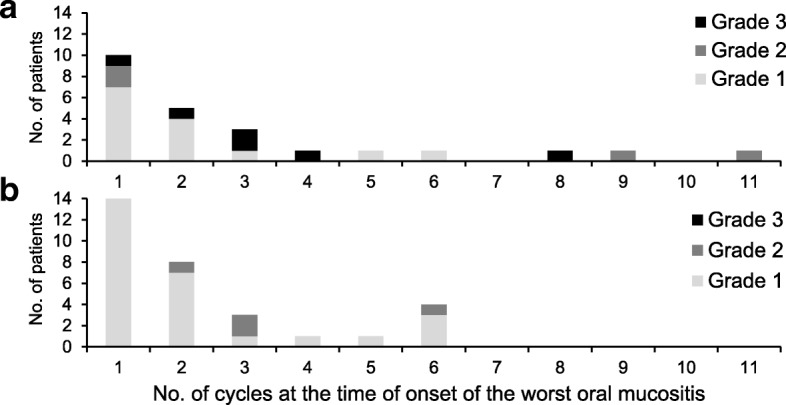
Secondary endpoint: the time to onset of the worst grade of oral mucositis each patient in the Pmab group (**a**) and Cmab group (**b**). The mean (SD) cycles to onset of the worst grade of oral mucositis each patient was 3.0 (2.9) in the Pmab group and 2.3 (1.7) in the Cmab group

**Fig. 3:**
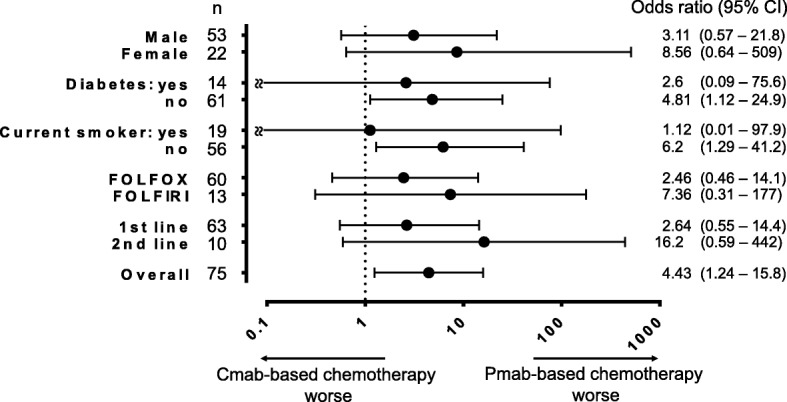
Odds ratio for grade 2–3 oral mucositis in pre-specified subgroups. Black dots indicate point estimates of the odds ratio and I bars indicate 95% confidence intervals of the odds ratio. The odds ratio was calculated by adding 0.5 to each value when no grade 2–3 oral mucositis was observed in each subgroup

The incidence of oral mucositis between the two groups is shown in Fig. 1. The primary outcome, the incidence of grade 2–3 oral mucositis, was significantly higher in the Pmab group than in the Cmab group (31.3% vs 9.3%, *p* < 0.05). Moreover, the incidence of grade 3 oral mucositis was significantly higher in the Pmab group than in the Cmab group (18.8% vs 0%, *p* < 0.01). Grade 2 to 3 oral mucositis in either groups was mainly characterized by glossitis (the tip of the tongue) and cheilitis (the inside of the lower lip). The time to onset of oral mucositis between the two groups is shown in Fig. 2. The mean cycles (SD) to the first onset of any grade of oral mucositis each patient was 1.8 (1.4) in the Pmab group and 2.2 (1.6) in the Cmab group (*p* = 0.32). The mean cycles (SD) to onset of the worst grade of oral mucositis each patient was 3.0 (2.9) in the Pmab group and 2.3 (1.7) in the Cmab group (*p* = 0.29). We also conducted subgroup analysis and calculated the odds ratio for grade 2–3 oral mucositis (Fig. 3). The point estimates of the odds ratio among subgroups were all poor for Pmab-based chemotherapy.

### Other toxicities of interest and TTF

**Fig. 4:**
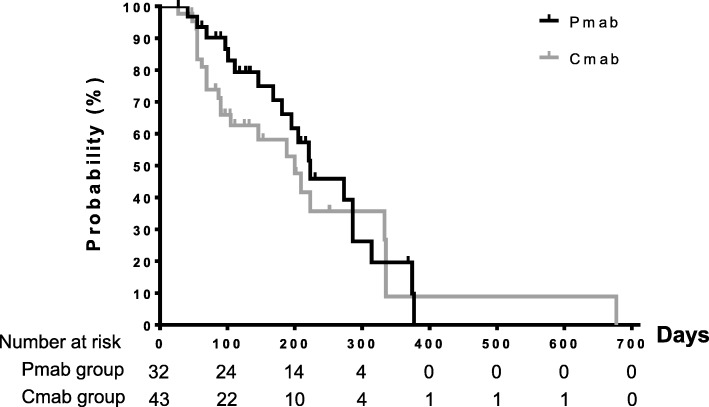
Time to treatment failure between the two groups. The Kaplan-Meier curve shows the time from treatment initiation to discontinuation for any reason between the two groups. The black line represents patients in the Pmab group and the gray line represents patients in the Cmab group. Tic marks mean censored cases. The median time to treatment failure were 223 days in the Pmab group and 200 days in the Cmab group (hazard ratio 0.78, 95% CI 0.42–1.38, *P* = 0.39)

The summary of other toxicities of interest is shown in Table 3. The incidence of toxicities between the two groups did not differ significantly. The median TTF was 223 days in the Pmab group and 200 days in the Cmab group (hazard ratio 0.78, 95% confidence interval 0.42–1.38) (Fig. 4).

**Table 3:** Summary of toxicities of interest

	Pmab group *N* = 32	Cmab group *N* = 43	*P*-value
Skin toxicity			
All grades	32 (100%)	41 (95%)	0.50
Grade 2-3	22 (69%)	32 (74%)	0.61
Grade 3	12 (38%)	11 (26%)	0.32
Hypomagnesaemia			
All grades	21 (66%)	27 (63%)	1.00
Grade 2-3	3 (9%)	3 (7%)	1.00
Neutropenia			
Grade 3-4	9 (28%)	16 (37%)	0.46
Grade 4	2 (6%)	3 (7%)	1.00

## Discussion

We clarified that Pmab-based chemotherapy concomitant with 5-FU could result in a higher incidence of grade 2–3 oral mucositis compared with Cmab-based chemotherapy concomitant with 5-FU. Furthermore, the odds ratio of grade 2–3 oral mucositis was higher in the Pmab group than in the Cmab group among subgroups. Conversely, other toxicities of interest and TTF were not different between the two groups. When these results of this study were compared with the previous studies, the following differences were found. First, in this study, grade 3 oral mucositis was not observed in the Cmab group. On the other hand, the previous MRC COIN study reported that the incidence of grade 3 or higher was 10% in patients treated with Cmab combined with FOLFOX [25]. As the mean half-life of Cmab in the steady state was reported to be 114 h (about 5 days) [29], the difference in oral toxicity between the MRC COIN study and our study may be due to the treatment interval of Cmab because the percentage of weekly Cmab administration was 100% in the MRC COIN study and 30% (Table 2) in this study. Second, our study reported a higher incidence (all grades: over 70%) of oral mucositis than previous studies (all grades: approximately 30%~ 40% [22–24]). We catch adverse events by carefully interviewing referring to the medical questionnaire answered by patient at each outpatient chemotherapy session. Therefore, we noted minor oral toxicity and oral pain, which resulted in the high incidence of oral mucositis. Third, although the previous study reported that the incidence of hypomagnesaemia was higher in patients treated with Pmab than in those with Cmab [20], the incidence of hypomagnesaemia did not differ between the two groups in this study. This may be because we administered prophylactic magnesium supplements at each cycle of chemotherapy after the occurrence of grade 1 hypomagnesaemia.

Anti-EGFR antibodies play a role extracellularly and not intracellularly because of their large molecular weight. Therefore, anti-EGFR antibodies mainly distribute in the blood and blood flow-rich tissues such as the kidneys, liver, spleen, and lung [30–32]. A previous study reported that the affinity to EGFR was higher for Pmab (50 pmol/L [33]) than for Cmab (400 pmol/L [34]). Based on this, toxicity in blood flow-rich tissues may likely occur with Pmab. Supporting this hypothesis, in the above mentioned ASPECCT trial, a randomised phase 3 trial that compared Pmab and Cmab in patients with chemotherapy-refractory WT KRAS exon 2 colorectal cancer, the incidence of grade 3–4 hypomagnesaemia was significantly higher in patients treated with Pmab than in patients treated with Cmab (7% vs 3%) [20]. As the kidneys are one of the most blood flow-rich tissues, anti-EGFR antibodies inhibit the renal distal tubule magnesium transporter, a transient receptor potential melastatin type 6 channel that is stimulated by EGF, resulting in hypomagnesaemia [35]. Therefore, due to the rich blood flow in the oral mucosa, the difference in oral toxicity between Pmab and Cmab may be explained by the same hypothesis. In addition, we observed grade 2–3 oral mucositis in both groups at the tip of the tongue and the inside of the lower lip, which are in contact with saliva. As salivary EGF plays an important role in the healing of damaged mucosa induced by radiotherapy [6, 7] and chemotherapy [8], mucotoxicity induced by anti-EGFR therapy combined with 5-FU may occur due to blockage of EGF at saliva-rich sites.

The strength of this study is that the incidence of oral mucositis induced by anti-EGFR antibody combined with 5-FU based chemotherapy was reported in the real world setting. At each outpatient chemotherapy session, we routinely assessed the severity of chemotherapy induced-toxicities based on CTCAE version 4.0, as well as toxicities that happened during chemotherapy intervals referring to the medical questionnaire answered by the patients. Therefore, regarding stomatitis, we meticulously interviewed and assessed about the oral condition when patients reported oral mucositis.

Some limitations exist in this study. First, we could not perform multivariate analysis for the primary endpoint considering covariates, such as history of diabetes and smoking status, because of the small sample size. Regarding neutropenia, which is a well-known confounder for oral mucositis, the incidence of grade 3–4 neutropenia was higher in the Cmab group. However, as the incidence of grade 2–3 oral mucositis was higher in the Pmab group, the incidence of neutropenia could not affect our study results. Second, as oral mucositis generally occurs during chemotherapy intervals, our assessment of oral toxicity mainly depended on patient interviews and patient diaries more so than the oral condition at the infusion date. Therefore, patient recall bias and interviewer bias cannot be excluded. To confirm our findings, a large prospective observational study should be conducted.

## Conclusions

Pmab-based chemotherapy resulted in significantly higher grades of oral mucositis compared with Cmab-based chemotherapy. The oral condition should be monitored carefully and early supportive care should be provided for patients treated with Pmab-based chemotherapy.

## Additional file

Additional file 1: **Table S1.** Doses and schedules of each 5-FU based chemotherapy combined with anti-EGFR antibody. (PDF 11 kb)

## Abbreviations

5-FU: 5-fluorouracil
Cmab: Cetuximab
CTCAE: The Common Terminology Criteria for Adverse Events
EGF: Epidermal growth factor
EGFR: Epidermal growth factor receptor
FOLFIRI: Infusional 5-fluorouracil plus leucovorin with the addition of irinotecan
FOLFOX: Infusional 5-fluorouracil plus leucovorin with the addition of oxaliplatin
LV: Leucovorin
Pmab: Panitumumab
TTF: Time to treatment failure
WT: Wild type
