# Supplementary material for: Oral mucositis associated with anti-EGFR therapy in colorectal cancer: single institutional retrospective cohort study
Source: BMC Cancer. 2018 Oct 5;18:957. doi: 10.1186/s12885-018-4862-z (PMC6173836; doi:10.1186/s12885-018-4862-z)
Supplement: Supplementary file 1 — Table S1. Doses and schedules of each 5-FU based chemotherapy combined with anti-EGFR antibody. (PDF 11 kb) [file 12885_2018_4862_MOESM1_ESM.pdf]

	Interval	Drug	Doses and schedules
Panitumumab + FOLFOX	q2w	Panitumumab<br>Oxaliplatin<br>L-leucovorin<br>5-fluorouracil	6 mg/kg on day 1<br>85 mg/m^2 on day 1<br>200 mg/m^2 on day 1<br>400 mg/m^2, 2400 mg/m^2 on day 1
Panitumumab + FOLFIRI	q2w	Panitumumab<br>Irinotecan<br>L-leucovorin<br>5-fluorouracil	6 mg/kg on day 1<br>150 mg/m^2 on day 1<br>200 mg/m^2 on day 1<br>400 mg/m^2, 2400 mg/m^2 on day 1
Panitumumab + LV5FU	q2w	Panitumumab<br>L-leucovorin<br>5-fluorouracil	6 mg/kg on day 1<br>200 mg/m^2 on day 1<br>400 mg/m^2, 2400 mg/m^2 on day 1
Weekly cetuximab + FOLFOX	q2w	Cetuximab<br><br><br>Oxaliplatin<br>L-leucovorin<br>5-fluorouracil	250 mg/m^2 on day 1, 8<br>(The initial dose was 400 mg/m^2 and the maintenance dose was 250 mg/m^2)<br>85 mg/m^2 on day 1<br>200 mg/m^2 on day 1<br>400 mg/m^2, 2400 mg/m^2 on day 1
Biweekly cetuximab + FOLFOX	q2w	Cetuximab<br>Oxaliplatin<br>L-leucovorin<br>5-fluorouracil	500 mg/m^2 on day 1<br>85 mg/m^2 on day 1<br>200 mg/m^2 on day 1<br>400 mg/m^2, 2400 mg/m^2 on day 1
Cetuximab + FOLFIRI	q2w	Cetuximab<br>Irinotecan<br>L-leucovorin<br>5-fluorouracil	500 mg/m^2 on day 1<br>150 mg/m^2 on day 1<br>200 mg/m^2 on day 1<br>400 mg/m^2, 2400 mg/m^2 on day 1
Cetuximab + LV5FU	q2w	Cetuximab<br>L-leucovorin<br>5-fluorouracil	500 mg/m^2 on day 1<br>200 mg/m^2 on day 1<br>400 mg/m^2, 2400 mg/m^2 on day 1

Table S1. Doses and schedules of each 5-FU based chemotherapy combined with anti-EGFR antibody.

5-fluorouracil was given as 400 mg/m^2 bolus infusion, then 2400 mg/m^2 over 46 hours continuous infusion. Abbreviation: q2w, given once every 2 weeks.
